# Propeptide-Mediated Inhibition of Cognate Gingipain Proteinases

**DOI:** 10.1371/journal.pone.0065447

**Published:** 2013-06-10

**Authors:** N. Laila Huq, Christine A. Seers, Elena C. Y. Toh, Stuart G. Dashper, Nada Slakeski, Lianyi Zhang, Brent R. Ward, Vincent Meuric, Dina Chen, Keith J. Cross, Eric C. Reynolds

**Affiliations:** Oral Health Cooperative Research Centre, Melbourne Dental School, Bio21 Institute of Molecular Science and Biotechnology, The University of Melbourne, Victoria, Australia; Russian Academy of Sciences, Institute for Biological Instrumentation, Russian Federation

## Abstract

*Porphyromonas gingivalis* is a major pathogen associated with chronic periodontitis. The organism’s cell-surface cysteine proteinases, the Arg-specific proteinases (RgpA, RgpB) and the Lys-specific proteinase (Kgp), which are known as gingipains have been implicated as major virulence factors. All three gingipain precursors contain a propeptide of around 200 amino acids in length that is removed during maturation. The aim of this study was to characterize the inhibitory potential of the Kgp and RgpB propeptides against the mature cognate enzymes. Mature Kgp was obtained from *P. gingivalis* mutant ECR368, which produces a recombinant Kgp with an ABM1 motif deleted from the catalytic domain (rKgp) that enables the otherwise membrane bound enzyme to dissociate from adhesins and be released. Mature RgpB was obtained from *P. gingivalis* HG66. Recombinant propeptides of Kgp and RgpB were produced in *Escherichia coli* and purified using nickel-affinity chromatography. The Kgp and RgpB propeptides displayed non-competitive inhibition kinetics with K_i_ values of 2.04 µM and 12 nM, respectively. Both propeptides exhibited selectivity towards their cognate proteinase. The specificity of both propeptides was demonstrated by their inability to inhibit caspase-3, a closely related cysteine protease, and papain that also has a relatively long propeptide. Both propeptides at 100 mg/L caused a 50% reduction of *P. gingivalis* growth in a protein-based medium. In summary, this study demonstrates that gingipain propeptides are capable of inhibiting their mature cognate proteinases.

## Introduction

*Porphyromonas gingivalis* is a major pathogen associated with chronic periodontitis. The organism’s cell surface cysteine proteinases, the Arg- and Lys-specific gingipains [1]–[2] have been implicated as major virulence factors that play an important role in colonisation and establishment of the bacterium as well as in the evasion of host defences [3]–[5].

Recent studies have demonstrated associations between periodontitis and systemic morbidities such as diabetes and cardiovascular disease [6], pre-term and low weight births [7], Alzheimer's disease [8], cancers [9], respiratory diseases [10] and rheumatoid arthritis [11]. The correlation between these systemic diseases and the entry of the bacterium and its gingipains into the circulation system are currently under investigation [12].

The gingipains RgpA, RgpB, and Kgp are encoded by three genes, *rgpA*, *rgpB,* and *kgp* respectively [13]–[15]. The gene *rgpB* encodes a single chain proteinase with a short 24 amino acid (aa) leader sequence, 205 aa propeptide, and a ∼500 aa catalytic domain [16]. In contrast, the longer *rgpA* and *kgp* genes each encode a leader sequence, propeptide, catalytic domain plus additional haemagglutinin-adhesin (HA) domains. Due to the importance of the gingipains in virulence [3]–[5] there is interest in the development of specific and safe inhibitors of the proteinases. Examination of the reported peptide-derived and non-peptide inhibitors of the gingipains in the literature reveals a surprising diversity of affinity, specificity and structural features. The inhibitors also display various modes of inhibition: competitive, non-competitive and uncompetitive [17]–[21]. To describe the specificity of proteases, a model of an active site composed of contiguous pockets termed subsites S1, S2 … etc is used with substrate residues P1, P2…etc occupying the corresponding subsites [22]. The residues in the substrate sequence are numbered consecutively outward from the cleavage site −P4−P3−P2−P1+P1'−P2'−P3'−P4'−, −S4−S3−S2−S1*S1'−S2'−S3'−S4'−. The scissile bond represented by the symbol+is located between the P1 and P1' positions, while the catalytic site is represented by the symbol *.

Bioinformatic analysis of known proteins and synthetic substrates cleaved by the gingipains reveals that although hydrophobic residues are frequently found at positions P4-P2 and P1’-P4’, overall the size, charge, and shape preferences of substrates are not clear (unpublished, [23]). This may reflect the ability of the gingipain active site to accommodate various substrates with only a strong specificity for an Arg or Lys residue in the P1 position.

Recent studies have highlighted that protease propeptides are a promising source of inhibitors for the cognate protease [24]–[25]. Many cysteine proteases are synthesized as inactive forms or zymogens with N-terminal propeptide regions. These propeptides may have multiple functions including inhibiting the proteolytic activity of the mature enzyme, folding of the precursor enzyme, protecting the enzyme against denaturation in extreme pH conditions, transporting the precursor enzyme to lysosomes, and mediating membrane association [26]. Typically the enzyme becomes activated upon removal of the propeptide by intra- or intermolecular proteolysis or in other cases by Ca^2+^ binding or acidification [26]. Although cysteine protease propeptides range from 30–250 aa, most are less than 100 aa residues [26]–[28]. The gingipain catalytic-domain propeptides are unusually long, being ∼200 residues suggesting that the gingipain propeptides may have a more complex function than the shorter propeptides of other proteases.

The aim of this study was to characterize the inhibitory potential of recombinant Kgp and RgpB propeptides against their cognate catalytic domains purified from *P. gingivalis*. The specificity of recombinantly expressed RgpB and Kgp propeptides for protease inhibition was determined as well as the interaction of the propeptides with both cognate and heterologous proteases and their effect on the growth of the bacterium.

## Methods

### Production of Recombinant Kgp Catalytic Domain

Plasmids and oligonucleotides used in the course of this work are listed in Table 1 and Table 2 respectively. Plasmids used were propagated in *Escherichia coli* α–Gold Select (Bioline Australia) or BL-21 (DE3) cells (Novagen). Allele exchange suicide plasmids (described below) were all linearised using XbaI restriction endonuclease (RE) digestion and transformed into electroporation-competent *P. gingivalis* cells [29] with transformants selected after anaerobic incubation at 37°C for up to ten days. EcoRV and ApaI recognition sequences were engineered into plasmid pNS1 [30] upstream of the *kgp* promoter [31] using oligonucleotide primers EA-For and EA-Rev (Table 1) and the QuikChange II Site-directed Mutagenesis Kit (Stratagene) following manufacturer’s instructions, generating pNS2. The *Bacteroides fragilis* cephalosporinase-coding gene *cepA* was amplified from a pEC474 template DNA [32] using oligonucleotides CepAf and CepAr and ligated into pGEM-T Easy (Promega) to generate pCS19. *cepA* was excised from pCS19 using EcoICRI/ApaI RE digestion and ligated into pNS2 that had been digested with BstEII (BII), end-filled then digested with ApaI. The resultant plasmid pPC1 has *cepA* that is transcribed from its own promoter and replaces nucleotides (nt) of pNS2 that include the *kgp* promoter and *kgp* nt coding from Met1-Tyr748. *P. gingivalis* W50 was transformed with pPC1 to produce the Kgp-null strain ECR364. Plasmid pPC2 was produced by ligating *ermF* excised from pAL30 [33] using ApaI and EcoRICRI RE digestion into pNS2 digested with ApaI and EcoRV. The nt coding ABM1 at the C-terminus of the Kgp catalytic domain (Gly681-A710, GEPSPYQPVSNLTATTQGQKVTLKWEAPSA) were then deleted from pPC2 using a combination of splicing by overlap extension (SOE) PCR, RE digestion and ligation as follows. Primer pairs ABM1del_For1 plus ABM1del_Rev1 and ABM1del_For2 plus ABM1del_Rev2 were used to generate two PCR amplicons which were annealed, extended and amplified using ABM1del_For1 and ABM1del_Rev2 as primers. The SOEn amplicon was digested with SnaBI and BstEII and ligated to SnaBI-BstEII digested pPC2 to generate pPC3 (Table 1) that was linearised and electroporated into ECR364 to replace *cepA* generating *P. gingivalis* ECR368 that produces rKgp with the ABM1 (Gly^681^– Ala^710^) deletion.

**Table 1:** Plasmids used in the course of this study.

Plasmids	Description^a^	Reference
pEC474	pBR322: : *cepA*	[32]
pCS19	pGEM-T Easy: : *cepA*	This study
pNS1	pUC18: : 3521 nt BamHI fragment of *P. gingivalis* W50 encompassing the 3′ of *PG1842* and the 5′ of *kgp*	[30]
pNS2	pNS1 with nucleotide T405C, A414G, T418C, A419C mutations to produce ApaI and EcoRV recognition sites.	This study
pPC1	pNS2: : *cepA. cepA* replaces nt that include the *kgp* promoter and *kgp* nt coding from Met^1^-Tyr^748^	This study
pPC2	*ermF* ligated between the ApaI and EcoRV sites of pNS2. *ermF* upstream of the *kgp* promoter.	
pPC3	pPC2 excluding *kgp* codons 681–710	This study
pET-28b	Expression vector	Novagen
pKgpPP1	Insert in pGEM-T Easy codes Kgp propeptide, residues 20–228	This study
pRgpPP1	Insert in pGEM-T Easy codes Rgp propeptide, residues 25–222	This study
pKgpPP2	Insert from pKgpPP1 in pET28b codes Kgp propeptide, residues 20–228	This study
pRgpPP2	Insert from pRgpPP1 in pET28b codes Kgp propeptide, residues 20–228	This study

**Table 2:** Oligonucleotides used in the course of this study.

Oligonucleotide	Sequence^a^
EA-For	GATTACAGTCGATATCTTGGCAAAGGGCCCATTGACAGCC
EA-Rev	GGCTGTCAATGGGCCCTTTGCCAAGATATCGACTGTAATC
CepAf	CGGATATAGGGACGTCAAAAGAG
CepAr	GGCTACAGATACTGGACGTCTCAA
ABM1del_For1	GCTTCTGCCGGTTCTTACGTAGC
ABM1del_Rev2	ACAAGAACTGGTAACCCGTATTGTCTC
ABM1del_Rev1	CTGCCTTCTTTACCTGAATTTGCTTGATCA
ABM1del_For2	AATTCAGGTAAAGAAGGCAGAAGGTTCCCG
Kgp-PP-for	ACGCAGCATATGCAAAGCGCCAAGATTAAGCTTGAT
Kgp-PP rev	ACGCAGCTCGAGtcaTCTATTGAAGAGCTGTTTATAAGC
Rgp-PP-for	ACGCAGCATATGCAGCCGGCAGAGCGCGGTCGCAAC
Rgp-PP-rev	ACGCAGCTCGAGtcaGCGCGTAGCTTCATAATTCATGAA

a Restriction endonuclease sites are underlined and stop codons in lowercase.

### Bacterial Strains and Growth Conditions

*P. gingivalis* W50, ECR368 producing rKgp, and strain HG66 [16] were grown at 37°C in a MACS MG500 anaerobe workstation (Don Whitely Scientific) with an atmosphere of 10% CO_2_, 5% H_2_, 85% N_2_, on 10% horse blood agar (HBA; Oxoid), with erythromycin supplementation (10 µg/mL) for ECR368. *P. gingivalis* was grown in batch planktonic culture in Brain Heart Infusion broth (BHI, 37 g/L), supplemented with haemin (5 mg/L), cysteine (0.5 g/L), and erythromycin (10 µg/mL) for ECR368. Culture purity was routinely assessed by Gram stain and observation of colony morphology on HBA plates.

*P. gingivalis* W50 was grown in a minimal medium [34]–[35] for at least 6 passages and then stored at −80°C for subsequent growth experiments. The minimal medium was prepared as follows: basal buffer (10 mM NaH_2_PO_4_, 10 mM KCl, and 10 mM MgCl_2_) was supplemented with haemoglobin (50 nM) and BSA (3% A-7906; Sigma-Aldrich Co.), pH 7.4, and filter sterilized (0.1 µm membrane filter Filtropur BT50, Sarstedt). The cells (10^8^ in 200 µL) were inoculated into each well of a 96-well microtitre plate (Greiner Bio-One 96-Well Cell Culture Plates) with 100 mg/L of rKgp-propeptide (Kgp-PP), rRgpB-propeptide (RgpB-PP) or Kgp-PP plus RgpB-PP. The plate was sealed with a plateseal microtitre plate sealer (Perkin Elmer Life Sciences, Rowville, VIC, Australia) and incubated overnight at 37°C in the anaerobic chamber. The cell density of the culture was monitored at 620 nm for 50 h at 37°C, using a Multiskan Ascent microplate reader (Thermo Electron Corporation). The *P. gingivalis* W50 isogenic triple mutant lacking RgpA, RgpB, and Kgp W50ABK [36] was used as a negative control of growth in the minimal medium.

### Purification of Kgp and RgpB

A procedure for the large scale purification of rKgp from the *P. gingivalis* strain ECR368 and RgpB from *P. gingivalis* HG66 was developed. Briefly, the bacteria were subcultured using a 1/100 v/v inoculum into 5–6 L BHI broth without additional haemin and incubated at 37°C for three days. The cells were pelleted by centrifugation (17,700 *g*, 60 min, 4°C) then the pH of the collected supernatant was lowered to pH 5.3 using acetic acid prior to filtration. The filtrate was concentrated using tangential flow filtration on a Sartorius Sartoflow alpha system with a 10,000 Da Molecular Weight Cut Off (MWCO) membrane, followed by diafiltration with 1 L 50 mM Na-acetate pH 5.3. The proteins were precipitated with chilled acetone added slowly to a final ratio of supernatant: acetone of 1∶1.5, and separated by centrifugation (17,700 *g*, 30 min, −10°C). The precipitate was solubilised in 50 mM Na-acetate pH 5.3 and centrifuged (17,700 *g*, 30 min, −10°C). The resultant supernatant was filtered through a 0.22 µm filter and desalted using Sephadex G-25 (200 mL) in 50 mM Na-acetate pH 5.3. The void volume was collected and then subjected to ion exchange chromatography using Q-sepharose (200 mL) equilibrated in 50 mM Na-acetate pH 5.3. After elution of the unbound fraction, a gradient of 0–1 M NaCl in 50 mM Na-acetate pH 5.3 was applied to elute the proteins containing Arg-protease activity and then remove the haemin.

The unbound fraction from the Q-sepharose, containing rKgp was diluted in 10 volumes of 50 mM Na-acetate pH 5.3 to reduce the ionic strength and loaded onto a 50 mL SP-sepharose column equilibrated in 10 mM Na-acetate pH 5.3. A gradient of 0–1 M NaCl in 50 mM Na-acetate pH 5.3, enabled the elution of the bound proteins that contained Lys-specific activity. The fractions were pooled, concentrated using 3,000 Da MWCO filters and subjected to size-exclusion chromatography using a 300 mL Superdex G75 column and the fraction containing rKgp was collected and stored at −70°C. Samples collected at each purification step were analysed for Lys- and Arg-protease activity, purity using SDS-PAGE, and protein estimation by absorbance at 280 nm, bicinchoninic acid (BCA) assay (Pierce, USA) and 2D Quant assay (GE Healthcare, Australia). The same protocol was used to purify RgpB from *P. gingivalis* HG66 culture supernatants. The concentrations of the RgpB (MW 55,636, 507 aa, ε = 54×10^3^ M^−1^ cm^−1^) and rKgp (MW 50,114 Da, 454 aa, ε = 105×10^3^ M^−1^ cm^−1^) were determined by measuring the absorbance at 280 nm using a 96 well UV plate and a PerkinElmer 1420 Multilabel Counter VICTOR3™ reader. The purified rKgp and RgpB proteins were subjected to trypsin hydrolysis and then LC-MS/MS analysis. The tryptic peptides were derived solely from the respective proteinase with no contamination by other proteins. The purified rKgp (0.66 U/mg) exhibited no Arg-X proteolytic activity and the purified RgpB (5 U/mg) exhibited no Lys-X proteolytic activity.

### Production and Purification of Recombinant Kgp and RgpB Propeptides

Recombinant Kgp and RgpB propeptides were produced with an N-terminal hexahistidine tag followed by the thrombin cleavage sequence to enable the binding to Ni-affinity resin with release following thrombin cleavage. DNA encoding the propeptide of *P. gingivalis* W50 Kgp (aa 20–228; O07442_PORGI) [30] or *P. gingivalis* W50 RgpB (aa 25–222; PG0506, CPG2_PORGI) [14] was amplified by PCR using the genomic DNA of strain W50 as a template and BIOTAQ DNA polymerase. Primer pair Kgp-PP-for and Kgp-PP-rev and primer pair Rgp-PP-for and Rgp-PP-rev, containing NdeI and XhoI RE sites and a stop codon in the antisense oligonucleotide were used for PCR of Kgp and Rgp propeptide coding DNAs respectively. The PCR products were ligated into pGEM-T Easy vector and the inserts sequenced. The plasmid inserts were then excised using NdeI and XhoI cleavage then ligated into NdeI/XhoI cleaved pET-28b expression vector (Novagen) and used to transform *E. coli* α-Gold Select cells. The recombinant plasmids were isolated and the insert was sequenced to verify correct amplification and ligation.

The recombinant pET-28b vectors were then transformed into *E. coli* BL-21 (DE3) (Novagen) and gene expression induced by addition of 1 mM isopropyl β-D-1-thiogalactopyranoside to cultures (OD_600__nm_ ∼0.5–0.7) growing in Luria-Bertani medium [37]. After 4 h of induced expression the cells were harvested by centrifugation (8,000 *g*, 20 min, 4°C), suspended in lysis buffer (50 mM Na_2_HPO_4_, 300 mM NaCl, 10 mM imidazole, pH 8.0) and disrupted by sonication (4 s on, 8 s off, 32% amplitude, for 15 min with a tapered 6.5 mm microtip) and stirring (30 min, 4°C). The lysate was centrifuged at 15,000 *g* for 15 min and the recombinant propeptides purified from the supernatant using Ni affinity chromatography with a modification of the procedure of Hondoh *et al.* (2006) [38]. Briefly, a 50% Ni-NTA (Qiagen) slurry (4 mL) was added to the supernatant, which was then stirred for 15 min at 4°C. The mixture was loaded on an open column with a volume of 20 mL and the flow through was removed. The resin was washed twice with 10 mL of purification buffer (50 mM Na_2_HPO_4_, 300 mM NaCl, 20 mM imidazole, pH 8.0). The column was stoppered and purification buffer (2 mL) containing 25 NIH units of thrombin (Sigma) was added to the slurry and incubated for 2 h at room temperature to cleave the propeptide His-tag and release propeptide from the nickel resin. The released propeptide and thrombin protease were then washed from the column using 15 mL of purification buffer and this solution was loaded onto a stoppered column containing 1 mL of Benzamidine Sepharose resin (Pharmacia). The solution was left to incubate for 15 min at room temperature to enable the thrombin protease to bind to the Benzamidine Sepharose resin. Once the flow through fraction was collected, the resin was washed twice with 2.5 mL of wash buffer (5 mM Na_2_HPO_4_, 50 mM NaCl, at pH 8.0) and each of the washes collected. The flow through fraction was then combined with the two wash fractions, resulting in a 20 mL solution. The extract was concentrated through a 3 kDa MWCO filter (Amicon) and applied to a gel filtration column (HiLoad 26/600 Superdex 75) attached to an AKTA-Basic FPLC system and eluted with 50 mM Tris-HCl, 150 mM NaCl, at pH 8.0 at a flow rate of 2 mL/min. The eluate was monitored at 280 and 215 nm. The eluate was collected, concentrated with a 3,000 MWCO Amicon centrifugal filter unit and the concentrations of the rKgp propeptide (MW 23,403 Da, 213 aa, ε = 11,920 M^−1^ cm^−1^) and the rRgpB propeptide (MW 23,204 Da, 209aa, ε = 10,430 M^−1^ cm^−1^) determined using absorbance at 280 nm.

### MALDI-TOF MS Analysis

Peptides and proteins were identified using an Ultraflex MALDI TOF/TOF Mass Spectrometer (MS) (Bruker, Bremen, Germany) and LC-MS. The samples were co-crystallized (1∶1 v/v) on an MTP Anchorchip™ 800/384 TF plate with saturated 4-hydroxy-α-cyanocinnamic acid matrix in standard buffer (97% acetone, 3% 0.1% TFA). The samples were analysed using Bruker Daltonics FlexAnalysis 2.4 and Bruker Daltonics BioTools 3.0 software with fragmentation spectra matched to an in-house *P. gingivalis* database installed on a local MASCOT server.

### In-gel Digestion and LC-MS Analysis

Protein bands were excised from the Coomassie® blue-stained SDS-PAGE gel, and analysed by LC-MS/MS as published previously [39]. The tryptic digests were acidified with trifluoroacetic acid (TFA) to 0.1% before online LC-MS/MS (UltiMate 3000 system, Dionex) with a precolumn of PepMap C18, 300 mm (inner diameter)×5 mm (Dionex) and an analytical column of PepMap C18, 180 mm (inner diameter) ×15 cm (Dionex). Buffer A was 2% (v/v) acetonitrile and 0.1% (v/v) formic acid in water and buffer B was 98% (v/v) acetonitrile and 0.1% (v/v) formic acid in water. Digested peptides (5 µL) were initially loaded and desalted on the precolumn in buffer A at a flow rate of 30 µL/min for 5 min. The peptides were eluted using a linear gradient of 0–40% buffer B for 35 min, followed by 40–100% buffer B for 5 min at a flow rate of 2 µL/min directly into the HCTultra ion trap mass spectrometer via a 50 mm ESI needle (Bruker Daltonics). The ion trap was operated in the positive ion mode at an MS scan speed of 8100 m/z/s over an m/z range of 200–2500 and a fast Ultra Scan of 26000 m/z/s for MS/MS analysis over an m/z range of 100–2800. The drying gas (N_2_) was set to 8–10 L/min and 300°C. The peptides were fragmented using auto-MS/MS with the SmartFrag option on up to five precursor ions between m/z 400–1200 for each MS scan. Proteins were identified by MS/MS ion search using Mascot v 2.2 (Matrix Science) queried against the *P. gingivalis* database obtained from J. Craig Venter Institute (JCVI.ORG).

### Intact Protein Analysis

An accurate molecular weight mass of the protein was determined using an Agilent 6220 Q-TOF by direct infusion Electrospray Ionization (ESI Q-TOF). The mass spectrometer was operated in positive MS only mode and data were collected from 100 to 2500 m/z. Internal, reference masses of 121.0508 and 922.0097 were used throughout. Deconvolution of the mass spectra was carried out using the Agilent Mass Hunter Qualitative Analysis software (B.05) and protein masses were obtained using maximum entropy deconvolution.

### Protease Inhibition Assays

Lys- and Arg-specific proteolytic activity was determined using the synthetic chromogenic substrates N-(*p*-tosyl)-Gly-Pro-Lys 4-nitroanilide acetate salt (GPKNA) and N-benzoyl-DL-arginine-4-nitroanilide hydrochloride (BapNA) (Sigma Aldrich), respectively. The protease assays were conducted as described previously [18]. The assay mixture contained *P. gingivalis* W50 whole cells (final cell density of ∼3×10^7^ cells/mL) or either the RgpB or rKgp (3.3–8.5 mg/L) proteases, recombinant propeptides at various concentrations, 1–10 mM cysteine pH 8.0, 5 mM dithiothreitol (DTT) and 1 mM chromogenic substrate made up to a final volume of 200 µL with TC150 buffer (50 mM Tris-HCl, 150 mM NaCl, 5 mM CaCl_2_, pH 8.0). Protease inhibitor, *N*α-*p*-tosyl-l-lysine chloromethylketone (TLCK) (1 mM) treated rKgp and RgpB proteases and blank wells with no proteases were used as negative controls. The caspase inhibitor Z-VAD-FMK (carbobenzoxy-valyl-alanyl-aspartyl-[O-methyl]- fluoromethylketone; Sigma USA) was used to selectively inhibit rKgp. Substrate cleavage was determined by measuring the absorbance at 405 nm at 10 s intervals for ∼20–30 min at 37°C using a PerkinElmer 1420 Multilabel Counter VICTOR3™. The proteolytic activity of the W50 whole cells and the RgpB/rKgp enriched fraction was determined as Units/10^11^ cells and Units/mg respectively, where 1 unit is equivalent to 1 µmole *p*-nitroanilide released/min.

RgpB and rKgp protease activity was also determined using DQ™ Green bovine serum albumin (DQ-BSA) (Molecular Probes, USA) [18], [40]–[41]. The assay mixture contained rKgp or RgpB (3.3–8.5 mg/L), recombinant propeptides (various concentrations), 1–10 mM cysteine, 5 mM DTT and DQ BSA (10 µL; 0.1 g/L), made up to a final volume of 200 µL with TC150 buffer. Negative controls were prepared as described earlier. The assay mixtures were incubated in the dark for 3 h at 37°C prior to measuring fluorescence using a fluorometer (PerkinElmer 1420 Multilabel Counter VICTOR3™).

Samples from each well were analysed for propeptide and protease hydrolysis using SDS-PAGE. Each sample (3×200 µL) was concentrated using a 3 kDa MWCO Amicon centrifugal filter unit at 14,000 *g* for 5 min. The concentrate was denatured using 5% (v/v) 1 M DTT and 25% (v/v) ×4 reducing sample buffer with heating for 10 min at 70°C unless otherwise stated. After microcentrifugation, 20–30 µL was loaded onto a precast 8–12% gradient Bis-Tris gel. SeeBlue® Pre-Stained standard was used as a molecular marker and a potential difference of 140 V and MES buffer (Life Technologies, Australia) were used to run the gel. The gel was stained with Coomassie® Brilliant Blue (G250) overnight and destained in deionised water.

### Propeptide Specificity

The specificity of each propeptide for its cognate enzyme, was examined by incubating the propeptide (50 mg/L) with the other gingipain at 7.5–8.5 mg/L concentration. The cross-reactivity with papain (P3375, Sigma) (2.75 g/L) was examined using BapNA as a substrate. The cross-reactivity with 60 Units of caspase (BML-SE169-5000, Enzo LifeSciences) was examined using (200 µM) Ac-DEVD-pNa (ALX-260-048-M005, Enzo LifeSciences) substrate.

### Determination of Type of Inhibition and Inhibition Constants

Inhibition kinetics were determined using purified rKgp (7.5 mg/L) and RgpB (8.5 mg/L) in the chromogenic substrate assay as described above. Initial reaction rates were obtained at substrate (GPKNA/BapNA) concentrations of 0.125, 0.25, 0.5, 0.75, and 1 mM and inhibitor (DTT-stabilised monomer of rKgp/rRgpB propeptide) concentrations of 0 to 200 mg/L. The proteolysis by rKgp (3.3 mg/L) was also examined using the fluorescent BSA substrate with rKgp propeptide concentrations of 2.5–50 mg/L. The initial rates of reaction were plotted against substrate concentrations. The curves were fitted individually by non-linear regression analysis to the Michaelis-Menten expression: v = d[P]]/dt = V_max_[S]/(K_m_+[S]) using the program Kaleidagraph (Synergy Software). The calculated K_m_ and V_max_ parameters of the proteolytic assays with increasing inhibitor concentrations were not consistent with competitive inhibition. Subsequently, the K_m_ value derived from the control experiment without inhibitor was fixed and used for all subsequent fitting of the data sets with increasing inhibitor concentrations. The reciprocal of the V_max_ values derived from the fitted curves were plotted against the inhibitor concentrations. K_i_ was obtained from the x-intercept value.

### Statistical Analysis

Protease activity data were subjected to a single factor analysis of variance (ANOVA). When the ANOVA indicated statistical significant difference (p<0.05) between the means of tested inhibitors, a modified Tukey test was performed on the data [42]–[43].

## Results

### Analysis of Proteinase Stability and Enzyme Kinetics

Both RgpB and rKgp were stable at 4°C at pH 5.3 for several months without loss of activity. The K_m_ for RgpB with the substrate BapNA was 64 µM and activation was dependent on the cysteine concentration in the proteolytic assay. Similar to RgpB the level of rKgp activation was dependent on cysteine concentration in the proteolytic assay and glycyl-glycine at 10 mM enhanced rKgp hydrolysis of the substrate GPKNA two-fold. The K_m_ value for rKgp was 46 µM consistent with the K_m_ value of 50 µM using the same substrate GPKNA, reported for Kgp isolated from *P. gingivalis* HG66 that releases the Lys-gingipain with associated adhesins into the culture fluid [44]. The K_cat_ was 4.5 s^−1^ and the K_cat_/K_m_ parameter representing the catalytic efficiency was 6.3×10^4^ M^−1^ s^−1^.

Since the synthetic small molecule chromogenic substrates are not the natural substrates *in* *vivo*, a fluorescently-labelled protein substrate, DQ-BSA with 23 arginines and 59 lysines was also used as a substrate to measure the proteolytic activity. Trypsin-like proteases cleave the self-quenched DQ-BSA releasing peptides with an average length of less than 8 amino acids [45]. Since the DQ-BSA is a multisite substrate the observed K_m_ is an average over all sites. Based on the equation below, the time course data were fitted to the expression with the assumption that the total product formed P_∞_ exactly equals S_0_ and where S<K_m_.
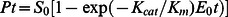



Using this assay with DQ-BSA as substrate the catalytic efficiency K_cat_/K_m_ for rKgp was 5.00×10^3^ M^1^ s^−1^ and for RgpB was 7.75×10^3^ M^−1^ s^−1^.

### Expression and Purification of Kgp and RgpB Recombinant Propeptides

**Figure 1:**
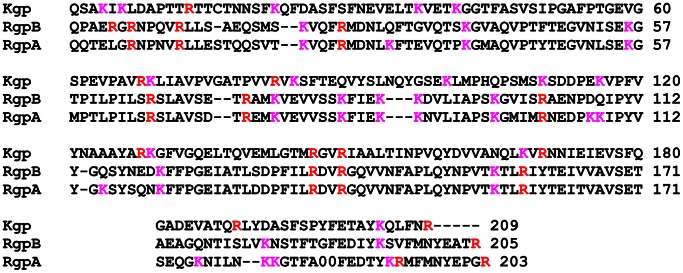
Cluster W 2.0.8 multiple sequence alignment of the Kgp, RgpB and RgpA propeptides. The distributions of the lysines (K) and arginines (R) are shown in pink and red respectively.

**Figure 2:**
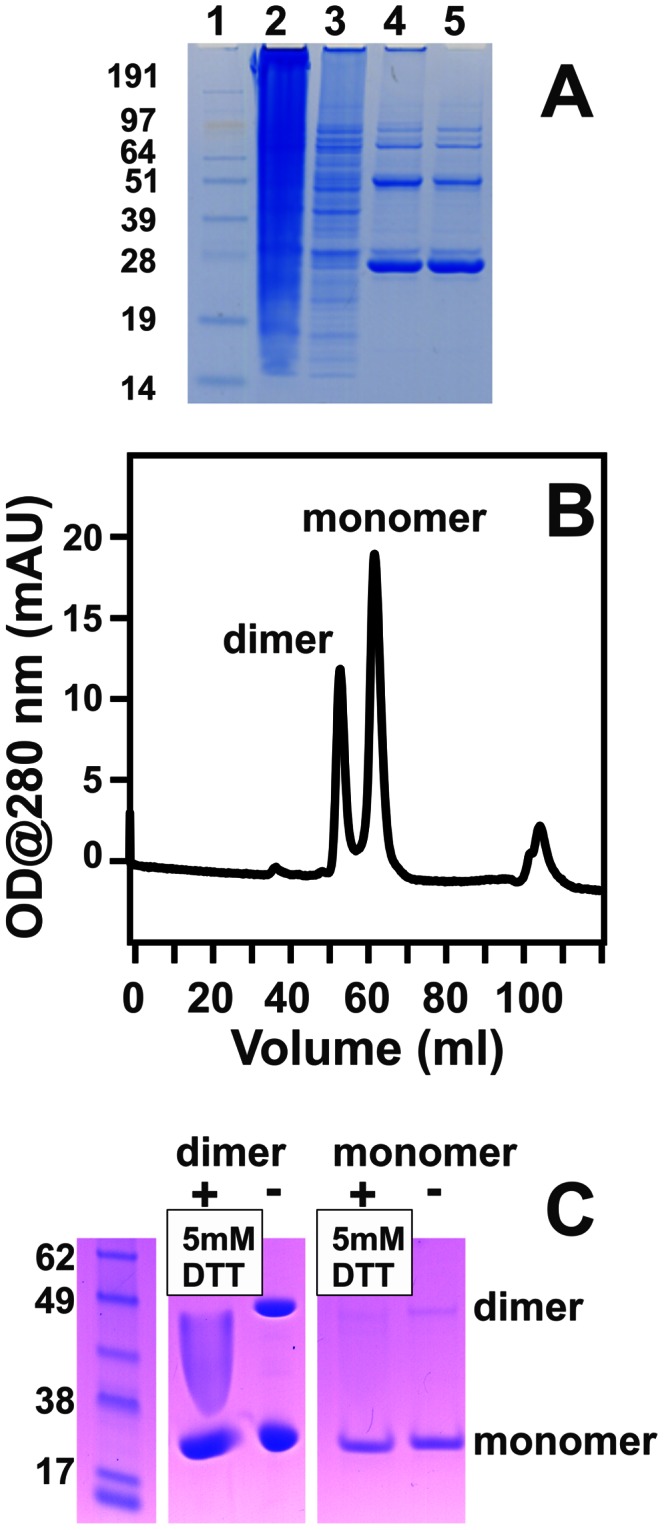
Purification of Kgp propeptide. (A) Non-reducing SDS-PAGE of recombinant Kgp propeptide expressed in *E. coli*. Lane 1: See-Blue® Pre-stained standard, where sizes in kDa are indicated; Lane 2: Flow through the Ni Column; Lane 3: Wash from Ni column; Lane 4: Thrombin cleaved product; Lane 5: Total thrombin-free Kgp propeptide extract prior to size exclusion chromatography. The gel was stained with Coomassie® Brilliant Blue (G250). (B) Size exclusion chromatogram (Superdex G75) of Kgp propeptide revealing dimerisation. (C) SDS-PAGE of the purified Kgp-propeptide monomer and dimer forms incubated with and without 5 mM DTT but without boiling revealing the disruption of the dimer with 5 mM DTT.

The Kgp and RgpB recombinant propeptides were designed to contain His-tag sequences followed by a thrombin cleavage site that was N-terminal to the mature propeptide sequence (Figure 1). The recombinant propeptides were expressed in *E. coli* and purified by binding the His-tagged propeptide to a nickel-sepharose affinity column, followed by thrombin cleavage to remove the His-tag and benzamidine-sepharose treatment to remove thrombin contamination. The purification of the Kgp propeptide is shown in Figure 2A. After size exclusion chromatography of the thrombin-cleaved recombinants, both Kgp and Rgp recombinant propeptides were deemed pure by SDS-PAGE and MS, with yields of 10–13 mg/L culture fluid. LC-MS analysis of tryptic peptides also confirmed the expected sequences of both propeptides. MS analysis using ESI Q-TOF showed a deconvoluted protein mass of 23,407.8, corresponding to a molecular mass of 23,403 Da for the thrombin cleaved Kgp propeptide and a deconvoluted protein mass of 23,205.3, corresponding to a molecular mass of 23,204 Da for the thrombin cleaved RgpB propeptide.

### Dimerisation of Kgp Propeptide

Initial studies with the Kgp-propeptide yielded inconsistent inhibition results. The Kgp propeptide exhibited the propensity to dimerize at higher concentrations as found in the cell lysate, Ni-affinity column-bound and thrombin-free products (Figure 2) and as detected by the relative K_av_ during size exclusion chromatography (Figure 2B). The monomer-dimer equilibrium at room temperature was evident for both Kgp propeptide monomer and dimer fractions as observed from the SDS gel within 1 h of separation by chromatography. The involvement of the single cysteine residue within the propeptide amino acid sequence in this dimerisation was investigated. Size-exclusion chromatography of the eluted dimer fractions incubated with 5 mM DTT demonstrated release of monomer. SDS-PAGE of the dimer and monomer fractions with and without 5 mM DTT confirmed the involvement of the cysteine residue (Figure 2C). Following the DQ-BSA substrate assay with the Kgp propeptide monomer and dimer in equilibrium, post-assay contents revealed that precipitation occurred on standing, suggestive of enzyme propeptide interactions. However the precipitation was not observed in the assays with added 5 mM DTT using the Kgp propeptide DTT-stabilized monomer. Iodoacetylation of the Kgp propeptide after DTT treatment prevented dimer formation based on Superdex G75 size-exclusion chromatography and non-reducing PAGE analysis.

Reproducible inhibitory activity was achieved with the monomer purified in the presence of 5 mM DTT using size-exclusion chromatography, with additional 5 mM DTT plus 10 mM cysteine in the proteolytic assays. These assay conditions ensured that the protease rKgp was fully reduced thus producing higher activity of the mature enzyme and a reproducible dose inhibitory response in both assays using DTT-stabilized monomer Kgp-propeptide. In the proteolytic assay with the chromogenic substrate, activity of rKgp (0.15 µM) increased by 49±1% with the addition of 5 mM DTT. In the DQ-BSA assay, addition of 5 mM DTT produced a 25±4% enhancement of activity.

### Propeptide Inhibition of Cognate Proteases

The inhibition of *P. gingivalis* W50 whole cell proteolytic activity by the Kgp and RgpB recombinant propeptides was determined using chromogenic substrates. The rate of substrate hydrolysis was monitored for linearity, to ensure there was no sharp increase in absorbance during the assay which would indicate that the inhibitory peptides were being used as a preferred substrate. The Kgp propeptide exhibited ∼35% inhibition of *P. gingivalis* W50 whole cell Lys-protease activity at 80 mg/L, while the RgpB propeptide exhibited 41% inhibition of W50 whole cell Arg-protease activity at 80 mg/L (Table 3).

**Table 3:** Summary of the proteolytic activities of various purified proteases and *P. gingivalis* whole cell preparations in the presence of RgpB and Kgp propeptides.

Protease	[Protease]	Inhibitor	[Inhibitor] (mg/L)	Substrate	% Proteolytic Activity
RgpB	0.0085 mg/mL	Kgp-PP	50	BapNA	105±3
Kgp	0.0075 mg/mL	RgpB-PP	50	GPKNA	118±11
Caspase 3	60 Units	Kgp-PP	100	Ac-DEVD-pNa (200 µM)	117±17
Caspase 3	60 Units	RgpB-PP	100	Ac-DEVD-pNa (200 µM)	133±15
Papain	2.75 mg/L	Kgp-PP	100	BapNA	102±10
Papain	2.75 mg/L	RgpB-PP	100	BapNA	103±17
Whole cell W50	3.2×10^7^ cells	Kgp-PP	40 80	GPKNA	92±23 65±29
Whole cell W50	3.2×10^7^ cells	RgpB-PP	40 80	BapNA	68±32 59±38

**Figure 3:**
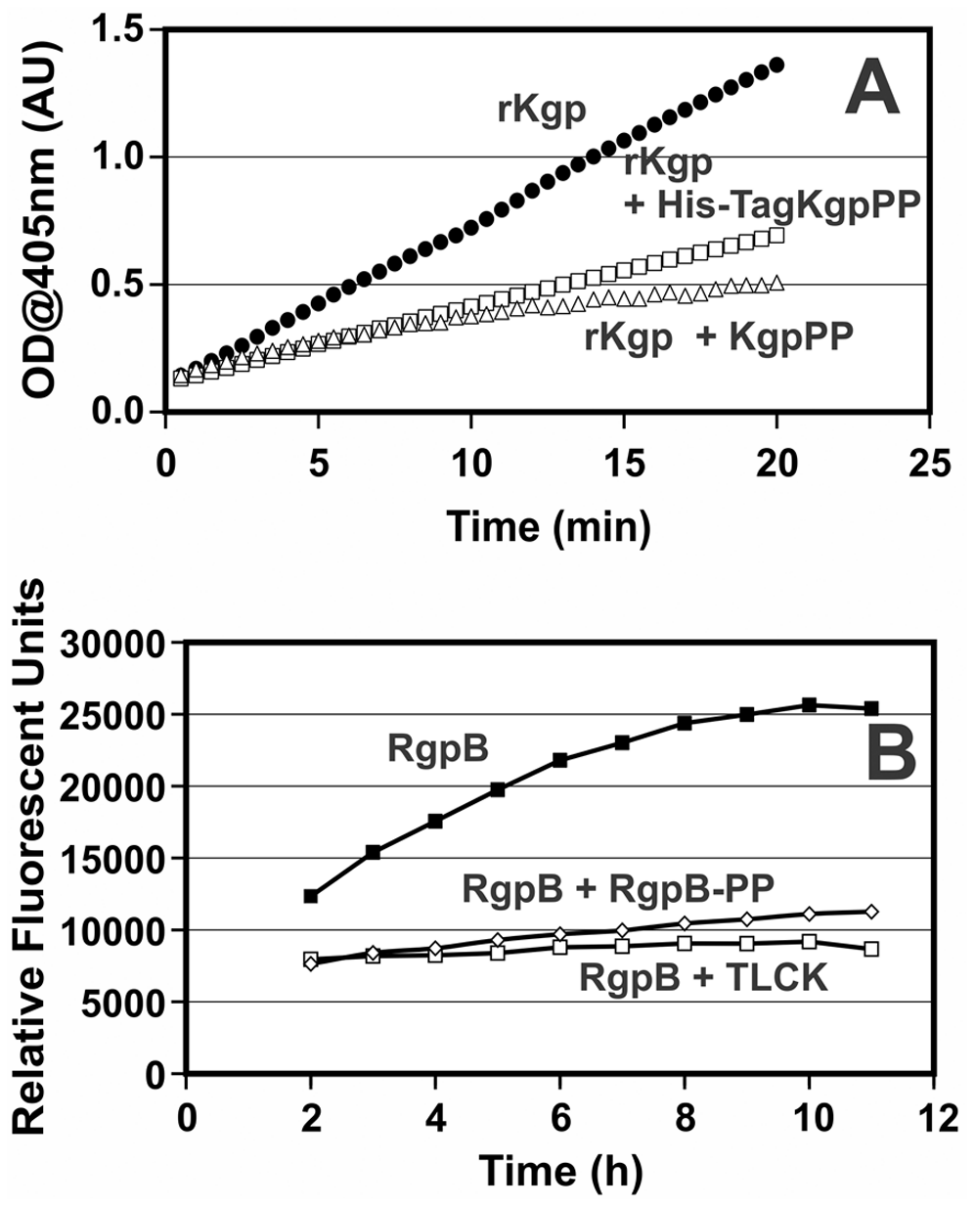
Proteolytic activity time course profiles of rKgp and RgpB using chromogenic and fluorescent substrates. (A) Time course of rKgp measured as change in absorbance (405 nm) without an inhibitor (•) and with 40 mg/L Kgp propeptide (Kgp-PP) with (□) and without the hexahistidine tag (▵) at 1 mM cysteine in the assay with the Lys-specific chromogenic substrate (GPKNA). The final concentration of rKgp per well is 1.16 mg/L. (B) Time course of RgpB using the fluorescent natural substrate DQ-BSA without RgpB-PP (▪), with 10 mg/L RgpB-PP (⋄) and 1 mM TLCK (□).

**Figure 4:**
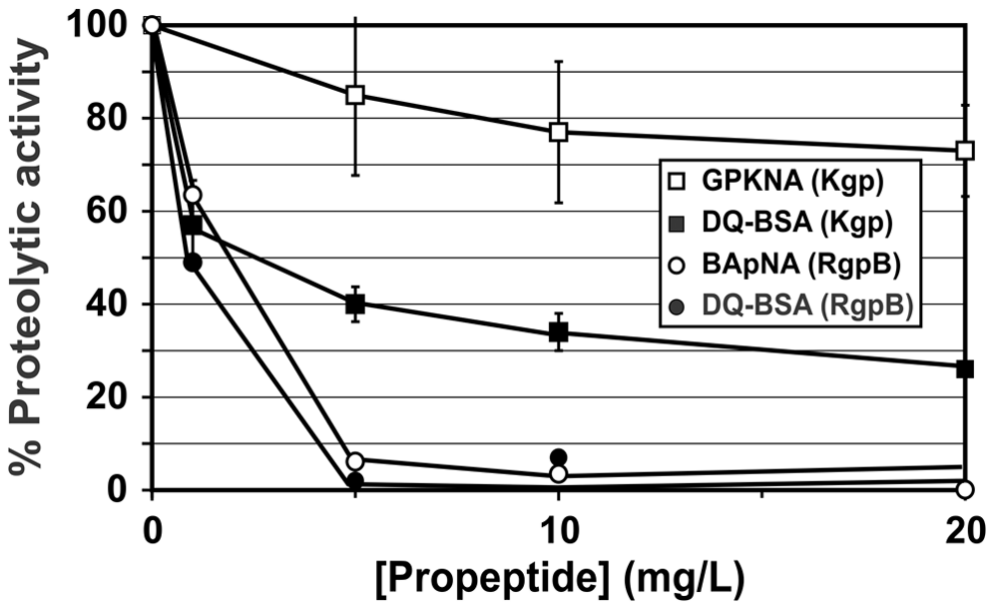
Inhibition of rKgp and RgpB by their propeptides. Assays were performed using the chromogenic substrates GPKNA (□) and BapNA (○). The % proteolytic activity was also determined using the fluorescent substrate DQ-BSA with rKgp (▪) and RgpB (•).

To establish targeted inhibition of the catalytic domain of the proteases, the propeptide was incubated with purified RgpB or rKgp. Using both chromogenic and fluorescent DQ-BSA assays, rKgp and RgpB were inhibited by their propeptides in a dose-dependent manner (Figures 3 and 4). The DTT-stabilized monomer at 100 mg/L (4 µM) demonstrated 68% inhibition of 0.15 µM rKgp compared to negligible 0–5% inhibition by the dimer with GPKNA as substrate. Similarly, in the DQ-BSA assay the DTT-stabilized monomer at 100 mg/L (4 µM) demonstrated 57% inhibition of 0.15 µM rKgp compared to negligible 0–4% inhibition by the equivalent dimer. The iodoacetylated monomer (100 mg/L) demonstrated 28±5% inhibition in the proteolytic assay using DQ-BSA as substrate. The RgpB recombinant propeptide at a concentration of 10 mg/mL inhibited ∼ 95% of RgpB activity.

The thrombin-like capability of the proteinases to cleave small molecule substrates while bound to inhibitors [46] was examined. The proteolysis assays with increasing concentrations of inhibitor were conducted with excess substrate. Fluorescence analysis of the 96-well plates 6–12 h after the proteolysis assay with DQ-BSA was consistent with the original inhibitor dose-response observed during the assay. In contrast the proteolysis assay using the small chromogenic substrates revealed that substrate consumption continued for a further 6–12 h irrespective of the presence and level of propeptide inhibitor. One interpretation for this observation is that the propeptide–protease interaction allowed small molecules to still have access to the active site, however larger substrates were blocked.

### Propeptide Selectivity and Specificity

Both RgpB and Kgp propeptides demonstrated selectivity for their own cognate protease with no inhibition observed when Kgp propeptides were incubated with RgpB and vice versa (Table 3). The specificity of the propeptides was further examined using two examples of cysteine proteases. The cysteine protease papain (2.75 mg/mL), with a propeptide of 115 residues, was not significantly inhibited by Kgp nor RgpB propeptides at 50 mg/L concentrations (Table 3). The cysteine protease caspase 3 that has structural homology with the RgpB and Kgp catalytic domains also was not inhibited by either Kgp or RgpB propeptides.

### Determination of Type of Inhibition and Inhibition Constants

**Figure 5:**
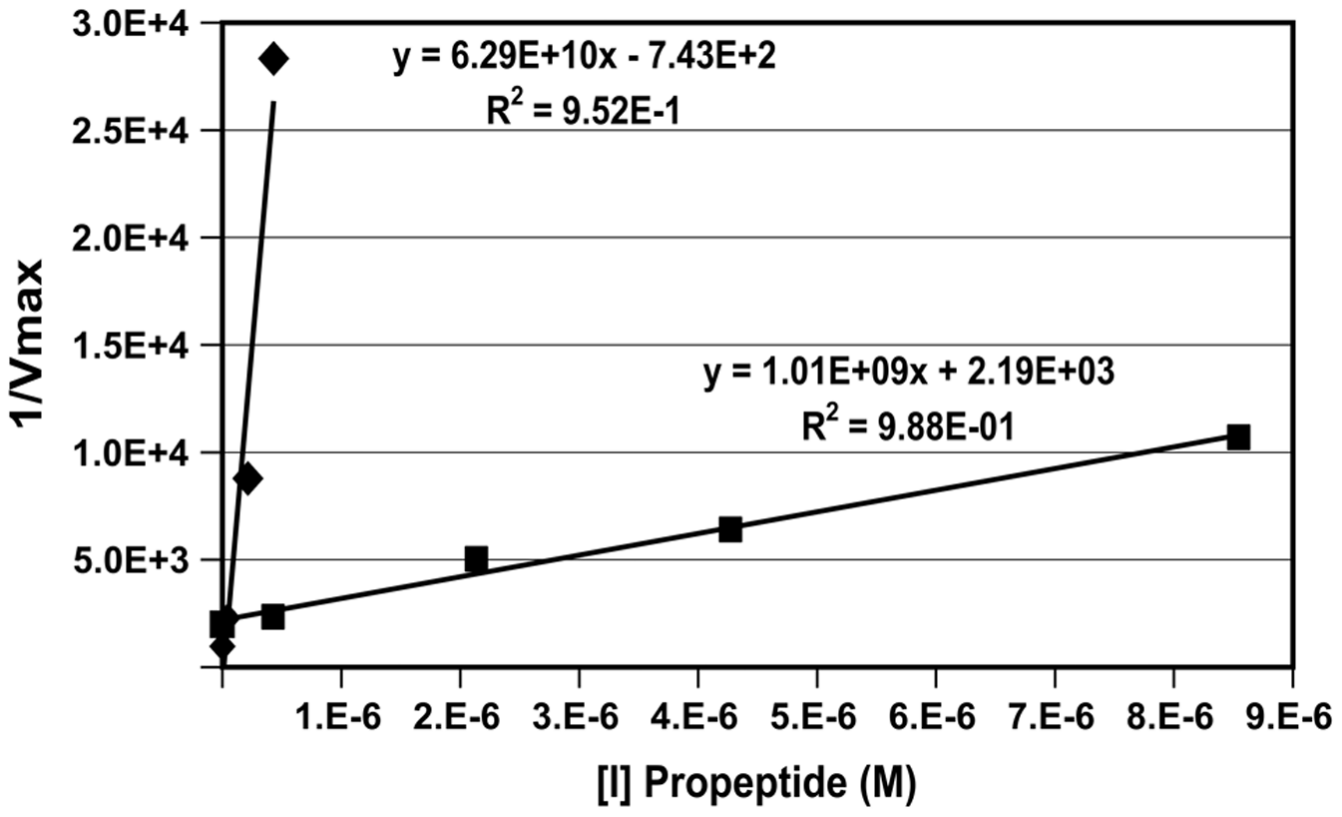
Characterisation of propeptide inhibition. Secondary plot of the reciprocal of V_max_ against inhibitor concentration ⧫ RgpB and ▪ rKgp. The K_i_’ values were obtained from the x-intercept.

In order to determine the inhibition constant of Kgp and RgpB propeptides and characterize inhibition mechanism, a kinetics analysis was performed with purified rKgp and RgpB. The dissociation constant K_i_^’^, for non-competitive binding of the inhibitor Kgp propeptide to the enzyme rKgp, was 2.01 µM for the monomer. The inhibition kinetics were also analysed for the fluorescent multi-site substrate DQ-BSA and the derived K_i_^’^ parameter was 2.04 µM. The RgpB propeptide also displayed non-competitive inhibition kinetics against RgpB with a K_i_^’^of 12 nM (Figure 5).

### Analysis of Propeptide Stability

The Kgp propeptide contains 13 Lys residues which could make the propeptide a potential substrate for Kgp proteolytic activity. To examine the fate of the Kgp recombinant propeptide in the presence of the proteases, the post-assay contents were analysed using SDS-PAGE and HPLC. The SDS-PAGE gels and HPLC chromatograms revealed intact Kgp and RgpB propeptides as well as degradation products that were then further analysed by LC-MS. Identification of the tryptic peptides coupled with the expected sizes of the Kgp propeptide fragments enabled a fragmentation pattern to be derived. Lys^110^ was the most susceptible to cleavage by the proteinase. Lys residues 4, 41, 69, 100, 129, 168 and 204 were also found to be susceptible to cleavage. In contrast, Lys residues 6, 22, 37, 84, and 116 were relatively resistant to proteolytic cleavage by Kgp. Kgp propeptide Arg residues at position 13, 146, and 149 were also observed to be relatively resistant to proteolysis by RgpB. The observation of Lys and Arg residues that are relatively proteolytically resistant to cleavage by Kgp and RgpB is indicative that the long propeptides have conformational preferences.

### *In vivo* Processing of Secreted rKgp Precursor Forms

**Figure 6:**
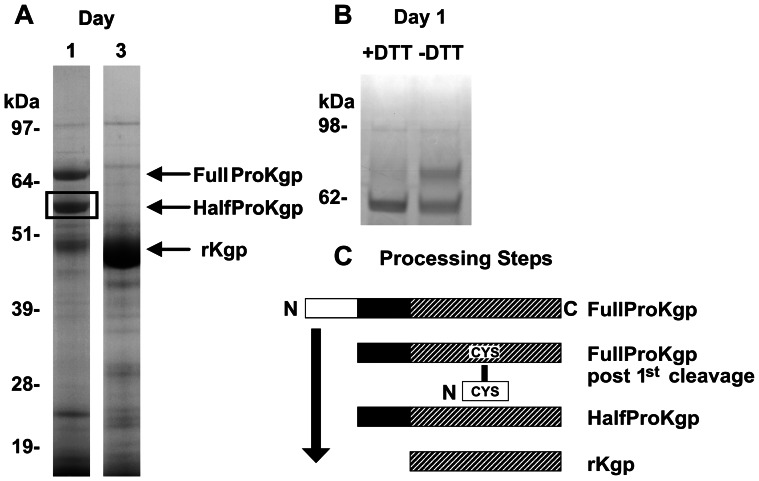
Processing of rKgp *in vivo.* (A) Reduced SDS-PAGE analysis of incompletely processed precursors observed in Day 1 and 3 culture supernatants of *P. gingivalis* ECR368 that releases rKgp. (B) SDS-PAGE analysis of the ∼60 kDa precursor form of rKgp with and without DTT revealing a single band under reducing conditions and two bands under non-reducing conditions, highlighting the disulphide bridge (CYS**–**CYS) that forms between the N-terminal half of the propeptide and the mature protease as summarized in (C) Processing steps: The N-terminal half of the propeptide is represented by the white rectangle, the C-terminal half by the black rectangle and the mature Kgp by the hatched rectangle.

A culture of *P. gingivalis* ECR368 was examined at Days 1 (exponential growth) and 3 (stationary phase) after inoculation. A reducing SDS-gel of the cell free culture fluid revealed the presence of precursors with estimated sizes of ∼70 and 60 kDa designated Full-ProKgp and Half-ProKgp respectively (Figure 6). These are consistent with precursor forms of the gingipains reported previously [47]–[48]. The ∼60 kDa intermediate present at equivalent or greater abundance indicates that the sequential cleavage rates k_2_<k_1_. The presence of an intra-molecular disulphide bond within the ∼60 kDa precursor form was investigated. A non-reducing SDS-gel (Figure 6) of the 60 kDa precursor revealed the presence of a higher molecular weight ∼70 kDa form, indicating that in a small population the 1st half of the propeptide although cleaved was still covalently attached to the Kgp catalytic domain through a disulphide bridge. The stable intermediate precursors with extra 10 kDa or 20 kDa propeptide regions eluted earlier than the mature Kgp as expected, from Superose 12 in 50 mM phosphate, 150 mM NaCl, pH 6.

### Propeptide-mediated Inhibition of *P. gingivalis* Growth

*P. gingivalis* W50 was grown in a protein-based minimal medium and reached a maximum cell density equivalent to an OD 620 nm of 0.32 after 40 h of incubation. The *P. gingivalis* triple gingipain mutant lacking RgpA, RgpB and Kgp does not grow in this defined protein-based minimal medium confirming that gingipain proteolytic activity is essential for the breakdown of the proteins (BSA and haemoglobin) in this medium. Both Kgp and RgpB propeptides demonstrated a significant inhibitory effect on *P. gingivalis* W50 growth in this protein-based minimal medium (Table 4).

**Table 4:** Relative growth inhibition of *P. gingivalis* in a protein-based minimal medium (MM) by RgB-propeptide (PP) and Kgp-propeptide (PP).

	Percentage of growth
MM	100%
MM+RgpB-PP 100 mg/L	55±19%
MM+Kgp-PP 100 mg/L	45±22%
MM+RgpB-PP 100 mg/L+ rKgp-PP 100 mg/L	60±12%

## Discussion

Despite recognition that the traversal of the Arg- and Lys- gingipains from the cytosol to the final cell surface destination is accomplished without premature activation, the role of the gingipain propeptides has not been extensively investigated. This current study has demonstrated that Kgp and RgpB propeptides inhibit the proteolytic activity of the membrane bound proteinases of *P. gingivalis* W50 in whole cell assays. To demonstrate targeted inhibition, characterise the mode of inhibition, and investigate the inter-molecular proteinase-propeptide interaction, cognate catalytic domains were purified from strains HG66 (RgpB) and ECR368 (rKgp).

In contrast to the nanomolar K_i_ estimated for the RgpB recombinant propeptide, a micromolar K_i_ was calculated for the Kgp propeptide. This has been attributed to the tendency of the Kgp propeptide to form covalent dimers through a single cysteine residue. The inhibitory capability of the mixture of monomer/dimer rKgp propeptides added to the proteolytic assay was inconsistent. This was resolved after separation of the DTT stabilized monomers from the non-inhibitory dimers using size-exclusion chromatography in 5 mM DTT.

The recent report of the RgpB propeptide co-crystallized with the cognate RgpB catalytic domain indicates that the propeptide attaches laterally to the RgpB catalytic domain through a large concave surface. The RgpB propeptide adopts an overall “croissant “ shape with a projecting “ inhibitory” loop consisting of sixteen residues (Lys113– Glu128) that approaches the active-site cleft of RgpB on its non-primed side in a substrate-like manner [49].

**Figure 7:**
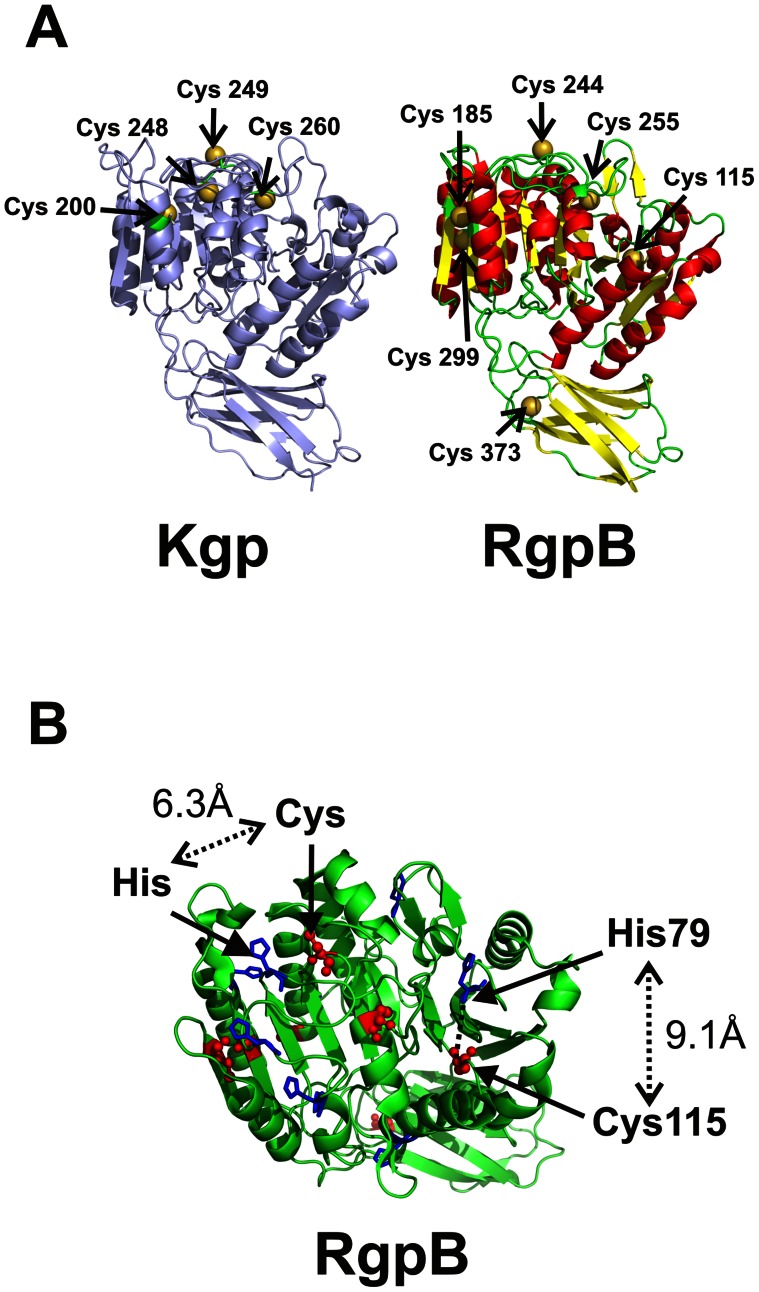
Current models of RgpB and Kgp. (A) Models of Kgp and RgpB based on the coordinates from PDB (1cvr.pdb), highlighting the cysteine residues. (B) Model of RgpB highlighting the His-Cys Cα distances in the two caspase sub-domains.

Observation of the precursor ProKgp (∼70 kDa) with the intermediate half-ProKgp (∼60 kDa) by reducing SDS-PAGE, at equivalent or greater abundance in the culture fluid during exponential growth of the *P. gingivalis* mutant ECR 368 indicates that the second cleavage step is slower than the first cleavage step. Although precursor forms have been observed for both RgpB and Kgp [47]–[48], the presence of the disulphide bridge between the Kgp propeptide and catalytic domain in the precursor form has not been reported previously and may have resulted from oxidation during extraction. This observation can not be explained by the reported structure of the RgpB propeptide interacting with the RgpB catalytic domain [49]. The catalytic domain of Kgp has four cysteines: Cys^200^, Cys^248^, Cys^249^ and Cys^260^ (Figure 7A). The observed *in vitro* inhibition by the discrete Kgp propeptide is not dependent on the formation of a disulphide bridge between the propeptide and catalytic domain as the inhibition is retained both in a reducing environment and by the iodoactylated Kgp propeptide. However disulphide bridge formation within the precursor form does occur in a non-reducing environment.

From a model of Kgp (Figure 7A) based on the RgpB structure, the catalytic cysteine is the most exposed and hence most likely to form a disulphide bond. The effect of the propeptide cysteine forming a disulphide bridge with either the catalytic cysteine Cys^249^ or the neighbouring Cys^248^ would have the effect of abolishing Lys-protease activity in the 70 kDa precursor form. This would be consistent with the recent report that an active site probe, a biotinylated irreversible Kgp-specific inhibitor [50] did not bind to the active site of the 70 kDa precursor form under non-reducing conditions [48]. However it is also plausible that the cleaved N-terminal half of the Kgp propeptide forms a disulphide bridge with one of the other two cysteines within the mature Kgp protease: Cys^200^ found only in Kgp, or Cys^260^, common to both RgpB and Kgp (Figure 7A). In the model of the mature proteinase, both these cysteines are less accessible for bridge formation; however, accessibility may be altered in the precursor form.

To understand the observed strong selectivity of the propeptides for the cognate proteases, the sequence variation of the RgpA/B and Kgp propeptides and the catalytic domains from the *P. gingivalis* strains W50, W83, ATCC 33277, TDC60, 381, W12 was examined. The RgpA/B and Kgp propeptides from the known *P. gingivalis* strains are all highly conserved with a calculated percentage identity (%ID) of 98–100% between the propeptide homologs. However sequence conservation is less between the RgpA and RgpB propeptide paralogs (75–76% ID ) and between the RgpA/B and Kgp propeptide paralogs (20–22% ID). Similarly the sequences of the catalytic domains of RgpA/B and Kgp are also highly conserved (94–100% ID) between the homologs with less conservation between the paralogs. This is consistent with the observed selectivity.

The specificity of the propeptides for the gingipains was examined using two examples of cysteine proteases. Since, the three gingipain propeptides range from 203 to 209 residues, significantly larger than the average propeptide lengths of ∼40 residues observed in most cysteine proteases [26], the 212 residue papain that is inhibited by its own 115 residue propeptide was selected. Neither Kgp nor RgpB propeptides demonstrated any inhibition for papain consistent with the differences between the papain and gingipain catalytic domains and active site configurations.

The second example was selected based on the structural similarities of the catalytic domains. Caspase 3 (pdb1pau) and RgpB structures (pdb1cvr) [51]–[53] share a common “caspase–hemoglobinase” fold with similar active site pockets despite limited sequence similarity [54]. The mature caspase 3 enzyme and zymogen backbone structures can be superimposed to within 3.8 Å over 106 residues. The current understanding of caspase activation and the caspase structure, presented a compelling argument to examine the effects of Kgp and RgpB propeptides on caspase activity. The absence of inhibition exhibited by both propeptides against caspase 3, highlights the specificities of the 200 residue propeptides.

Both RgpB and, by homology, Kgp catalytic domains have the appearance of two adjacent caspase sub-domains plus the C-terminal Ig-fold [54]. The RgpB active site cysteine and histidine occur in the second caspase sub-domain and their respective Cα atoms are within 6.3 Å. In the first caspase sub-domain the RgpA/RgpB sequences have a cysteine (Cys^115^) and histidine (His^79^) at topologically analogous positions (Figure 7B). The catalytic potential of these two residues in RgpB and RgpA has not been explored. However, this difference between Kgp and RgpB/RgpA may also account for the selectivity exhibited by the cognate propeptides.

**Figure 8:**
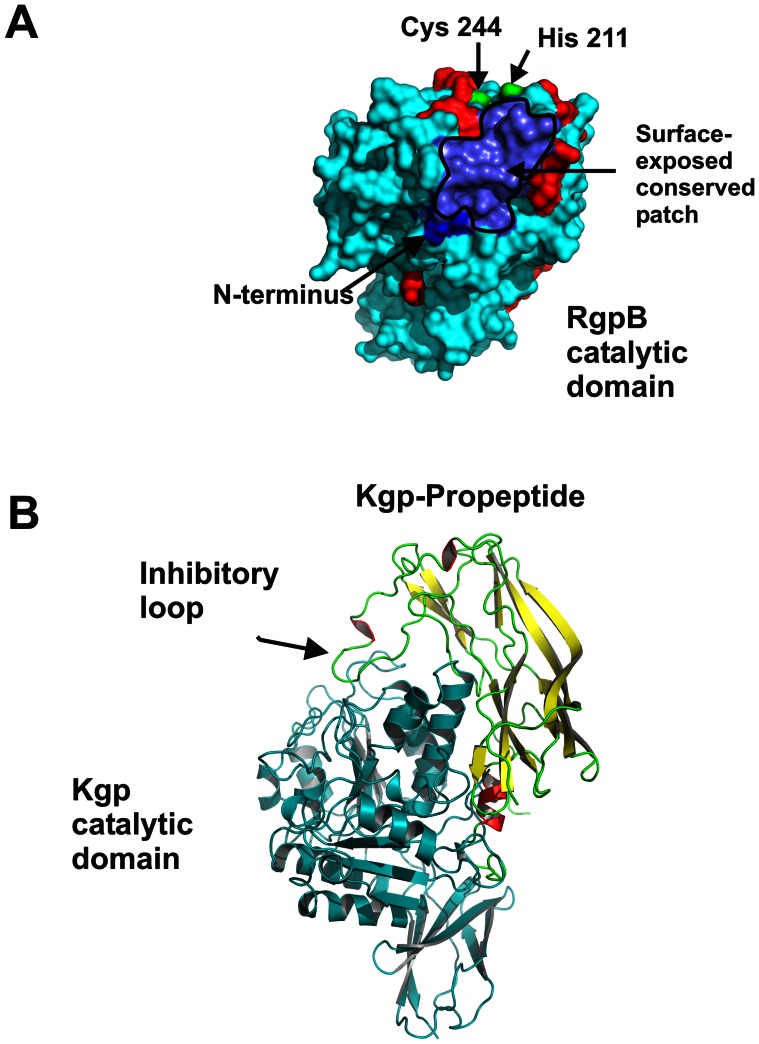
Interaction of gingipain catalytic domains with their propeptides. (**A**) Model of RgpB highlighting the N-terminus, catalytic Cys and His residues, and residues that differ between strains in red. The residues that form a surface-exposed conserved patch are predicted to interact with the propeptide. (**B**) Schematic representation of the inhibition of Kgp by its propeptide. Kgp was modelled using Orchestrar from within Sybyl-8.1 [55] and based on the X-ray crystal structure of RgpB 1cvr.pdb [56]. The propeptide is based on the A chain of the X-ray crystal structure of RgpB interacting with its propeptide 4ief.pdb [49].

To further understand the interaction between the conserved propeptides and the cognate proteases, the residues within the catalytic domains of RgpA, RgpB and Kgp that differ between the different strains of *P. gingivalis* were identified. These point mutated residues found within RgpA and RgpB were mapped against the crystal structure of RgpB. This revealed that the residues located on the first α-helix immediately C-terminal of the fifth β-strand and the N-terminal portion of the next α-helix are conserved. This surface-exposed, conserved patch is depicted between the position of the known N-terminal residue of the catalytic domain and the active site (Figure 8A). In the case of Kgp, 28 residues that differ between different strains of *P. gingivalis* were identified. Three residues within 10 Å of the catalytic site were changed: A449S, L454S, and I478V. Interestingly, the A449S and L454S point mutations are found together in F5XB86 (TDC60), Q51817 (W83), and Q6Q4T4 (an un-named strain) making a small region close to the catalytic site of Kgp more hydrophilic in those strains. Mapping all the 28 point-mutated residues to the model of Kgp revealed an analogous surface-exposed, structurally identical, conserved patch in Kgp. The surface-exposed, conserved patches in RgpB and Kgp are predicted to be covered by the propeptide in the respective zymogens.

Models of both Kgp and the Kgp propeptide were produced using Orchestrar from within Sybyl-8.1 [55] and based on the X-ray crystal structure of RgpB (1cvr.pdb) [56] and chain A from the crystal structure of RgpB co-crystallized with its propeptide (4ief.pdb) [49] respectively. The Kgp propeptide model was validated by calculating the ‘Fugue alignment’ [57] between the Kgp and RgpB propeptides, which gave a Z-score of 10.72 classified as ‘certain’ with greater than 99% confidence. The model of the propeptide had an rms deviation of 1.28 Å from the crystal coordinates after energy minimization to a maximum gradient of 0.5 kcal mol^−1^ Å^−1^ using the AMBER force-field. A model of the Kgp propeptide docked with Kgp was then produced by independently aligning by least-squares the model of Kgp and the model Kgp propeptide against the B and A-chains respectively of the co-crystallized RgpB/RgpB propeptide (4ief.pdb). This alignment predicts that the Lys^110^ of the inhibitory-loop of the Kgp propeptide will insert into the catalytic pocket of Kgp.

A schematic representation of the inhibition of Kgp by its propeptide based on this model is shown in Figure 8B. From the model structure the cleavage of the propeptide at Lys^110^ will leave a substantial protein domain still capable of allosterically blocking access to the catalytic site by large, substrate proteins. The bound orientation of the propeptides with their proteases is consistent with an interaction between the identified conserved patch (Fig. 8A) and the propeptide. The schematic (Fig. 8B) is also consistent with possible exosite binding that could explain the selectivity and specificity of the propeptides. Experimentally, the peptide bond C-terminal to Lys^110^ was found to be susceptible to cleavage by Kgp. This is consistent with the location of Lys^110^ being in a loop; the peptide bond is only protected from cleavage when the propeptide is bound to Kgp with the appropriate orientation.

It was interesting to examine the effects of the propeptides on growth of *P. gingivalis*. The requirement of cell surface located proteinases for nutrient acquisition, tested using the triple mutant without the RgpA, RgpB and Kgp gingipains in a protein-based minimal medium was consistent with previous reports [41], [58]. The observed retardation of the planktonic growth of *P. gingivalis* by the added propeptides highlights their potential for inhibition of *P. gingivalis* growth and virulence.

In summary the *P. gingivalis* cell surface gingipains are carefully regulated prior to activation by high-selectivity propeptides that are tailored to each proteinase. It is possible that the long propeptide has a role in propeptide-mediated folding as well as preventing proteinase premature activation throughout the multiple processing, propeptide detachment, and rearrangement events that occur to enable the cell surface assembly of the gingipain complexes.
